# Testing Agents for Prevention or Reversal of Type 1 Diabetes in Rodents

**DOI:** 10.1371/journal.pone.0072989

**Published:** 2013-08-30

**Authors:** Christian W. Grant, Catherine M. Moran-Paul, Shane K. Duclos, Dennis L. Guberski, Guillermo Arreaza-Rubín, Lisa M. Spain

**Affiliations:** 1 National Institute of Diabetes and Digestive and Kidney Diseases (NIDDK), National Institutes of Health (NIH), Bethesda, Maryland, United States of America; 2 Biomedical Research Models (BRM), Inc., Worcester and Springfield, Massachusetts, United States of America; Children's Hospital Boston/Harvard Medical School, United States of America

## Abstract

We report the results of an independent laboratory’s tests of novel agents to prevent or reverse type 1 diabetes (T1D) in the non-obese diabetic (NOD) mouse, BioBreeding diabetes prone (BBDP) rat, and multiple autoimmune disease prone (MAD) rat models. Methods were developed to better mimic human clinical trials, including: prescreening, randomization, blinding, and improved glycemic care of the animals. Agents were suggested by the research community in an open call for proposals, and selected for testing by an NIDDK appointed independent review panel. Agents selected for testing to prevent diabetes at later stages of progression in a rodent model were a STAT4 antagonist (DT22669), alpha_1_ anti-trypsin (Aralast NP), celastrol (a natural product with anti-inflammatory properties), and a Macrophage Inflammatory Factor inhibitor (ISO-092). Agents tested for reversal of established T1D in rodent models were: alpha_1_ anti-trypsin (Aralast NP), tolerogenic peptides (Tregitopes), and a long-acting formulation of GLP-1 (PGC-GLP-1). None of these agents were seen to prevent or reverse type 1 diabetes, while the positive control interventions were effective: anti-CD3 treatment provided disease reversal in the NOD mouse, dexamethasone prevented T1D induction in the MAD rat, and cyclosporin prevented T1D in the BBDP rat. For some tested agents, details of previous formulation, delivery, or dosing, as well as laboratory procedure, availability of reagents and experimental design, could have impacted our ability to confirm prior reports of efficacy in preclinical animal models. In addition, the testing protocols utilized here provided detection of effects in a range commonly used in placebo controlled clinical trials (for example, 50% effect size), and thus may have been underpowered to observe more limited effects. That said, we believe the results compiled here, showing good control and repeatability, confirm the feasibility of screening diverse test agents in an independent laboratory.

## Introduction

The NOD mouse was discovered in 1980 [1], and the BBDP rat in 1974 [2]. The MAD rat, (formerly LEW.1WR1) was reported as an inducible model in 2005 [3]. Since then these rodents been used as models of autoimmune destruction of insulin-producing beta cells. The parallels between the human disease and the rodent models, especially the high degree of correspondence of genetic susceptibility determinants, are clear. A major limitation of the effort to model the human disease using the NOD mouse or BB rat is the lack of understanding of the human disease. We simply cannot know which features of the rodent diseases are relevant until we know the important players in human pathogenesis. Moreover, the ability of the rodents to model the human disorder in terms of responses to therapy, especially immune-modulatory interventions, have been called into question [4]. Correlative or descriptive studies using human specimens from clinical research provide hints as to which mechanisms might be contributing to pathogenesis and to therapeutic responses in humans.

Given the broad evolutionary conservation of immune mechanisms between rodents and humans, a well-understood and properly applied rodent model is useful in providing tools for studying possible pathogenic mechanisms and therapeutic interventions. Many studies have demonstrated efficacy in the prevention and/or reversal [4,5,6,7,8,9,10,11,12,13,14,15,16,17,18,19,20,21,22,23,24,25] of spontaneous T1D in rodent models. Noting the many differences between rodents and humans, it is also not surprising that some of these mechanisms operating in rodents may ultimately fail in human clinical trials for T1D prevention or reversal. It is also expected that some mechanisms important for human disease will be missed if the particular rodent models, e.g., the NOD mouse, is exclusively relied upon, or conversely, that enthusiasm for interventions with real therapeutic potential in humans may be dampened by poor results in a rodent study. Neither of these caveats justifies abandoning rodent models, but they must be acknowledged.

In addition to the limitations imposed by the differences in biology of rodents and humans, methodological factors also play a part in determining the usefulness of animal models. As reported by Landis and colleagues, a systematic review and meta-analysis of published animal studies revealed that inadequate reporting correlated with failures of rodent models to predict clinical success [26]. For example, deficient methodological reporting correlated with overstated findings, while studies that reported randomization and blinding of animal experiments tended to show smaller effect sizes of tested agents. This suggests that methodological flaws in animal model studies could be common, and suggests that the animal models literature should be cautiously approached. The aim of each animal study and its methods must be carefully considered when interpreting the power of the results for predicting clinical trial success. This is an important problem because animal studies often provide part of the preclinical data used to justify clinical trials, and if they are inadequate, they might add to unnecessary risks for subjects, and waste research resources. Of course, a thorough review of available and relevant animal data is a responsibility shared by translational researchers and regulatory agencies.

We attempted to address the issue of consistency in study design, reporting, and interpretation of results by funding an independent testing laboratory using internally standardized and validated methods for preclinical testing of potential therapies in the spontaneous NOD mouse and BB rat models. We established the testing program with the intent to publish all results, whether positive or negative, hoping to reduce publication bias in favor of “positive” findings, and to make study results freely available to the research community. Scientists developing agents may not have access to the most appropriate animal models for efficacy testing in T1D. To fill this gap, NIDDK established a contract with Biomedical Research Models, Inc., (BRM, Inc.) to provide preclinical testing of agents for efficacy in reversing or preventing T1D in rodents. NIDDK issued a Request for Information that provided a pathway for scientists to propose agents for testing. Proposed agents were reviewed for program applicability and translational potential by an independent review committee. In this initial stage of the testing program, we emphasized testing of agents for which there was some prior data for efficacy, albeit incomplete or preliminary, reported in the literature or communicated by the agent provider. In addition, we selected agents that were known to react with both rodent and human targets, with similar biological responses and functional pathways, with the expectation that such agents could be more directly translated to the clinic. For a positive control, we used anti-CD3 which is a mouse-specific equivalent of the human therapeutic. Meanwhile, BRM established significantly improved insulin therapy methods for rat and mouse models of spontaneous autoimmune diabetes [27]. These methods provide improved glycemic control in the animals as soon as possible after disease onset, to better model human therapy and to allow rigorous testing of agents for diabetes reversal.

## Materials and Methods

### Randomization

Animals were not randomized into treatment groups; instead we used a staggered and fixed enrollment onto study, i.e., 1^st^ diabetic animal into Group 1, 2^nd^ diabetic animal into Group 2, etc. Data was not collected randomly, but according to a predetermined (protocol-specific) schedule.

Blinding:

1) Allocation concealment: Staff were not blinded as to which group would be enrolled next since a fixed staggered enrollment procedure was used.
2) Assessment of outcomes: Staff were not blinded as to the treatment groups of animals when making blood glucose measurements and general health assessments. Staff and subcontractors were blinded to treatment group when reading slides during histological assessments.
3) Interim data analysis: interim analyses were performed, but these did not alter the number of animals in the study, i.e enrollment was pre-determined and was not reduced or increased as a result of interim analyses.

### Sample size estimation

We used either an independent biostatistician or Statmate (Graphpad Software) to calculate sample sizes before each study. The analyses were predetermined (protocol-specific) and not adjusted at the study end.

### Data handling

Data collection was protocol specified and all data was analyzed. Outliers were not excluded. Primary end points were protocol-specified and finalized prior to initiation of each study. Attrition (e.g., diabetes onset in prevention studies and humane endpoints [unresolved hyperglycemia or body weight loss] for reversal studies) was reported in the study figures, tables, and reports. Pseudo replicates (technical replicates – analyzing the same sample over and over) were not performed. Significant protocol or procedural deviations were documented and reported. In some circumstances, we performed a few studies with different designs to confirm or expand upon earlier results.

### Test Articles and Formulation

Cyclosporin A and 20% intralipid were purchased from Sigma, formulated once weekly and stored at 2-8°C. DT22669 was provided by the manufacturer (DiaKine), stored at 2-8°C, and formulated in sterile 0.9% sodium chloride. Aralast NP was purchased from the manufacturer (Baxter Healthcare), and stored at 2-8°C until formulated per manufacturer’s instructions within three hours of administration. ISO-092 was provided by the Feinstein Institute for Medical Research, resuspended in vehicle and stored ambient during the course of the study. Celastrol was purchased from the manufacturer (Pi & Pi Technology) in single-use vials and stored at 2-8°C until formulated on the day of administration. PGC-GLP-1 (and PGC control) were provided by PharmaIN Corporation in septum vials, resuspended as needed with sterile 0.9% sodium chloride, and stored for up to three weeks at 2-8°C. PGC stands for protected graft copolymer made up of 15-30 kDa polylysine in which 55% of epsilon amino groups linked to 5kDa Methoxy polyethylene glycol and the remaining amino groups linked with stearic acid (31). The pharmacokinetic behavior PGC-GLP-1 formulation was tested in mice prior to shipment as part of several quality control processes and the results were consistent with the paper cited above. Tregitope peptides and liposomes were provided as concentrated stock solutions at -20°C or 2-8°C, respectively, which were diluted and mixed in the appropriate combinations on each day of administration. Hamster anti-mouse CD3 IgG F(ab’)2 (clone 145-2C11, BioXcell) or isotype control (hamster IgG F[ab’]2) was screened for prevalent rodent infectious agents (Charles River Laboratories), and aliquoted into single use vials. Aliquots were thawed as needed and diluted in sterile 1x PBS on the day of administration.

### Animals

Animals were group housed (n=2-4/cage) in polycarbonate cages with wire lids and filter covers during study except for when a single animal remained in a group or during post-operative recovery (i.e., subcutaneous pump implantation), provided autoclaved 7012 (rats) or irradiated 5LG4 (mice) diet and filtered tap water or spring water (for MAD rats studies in isolators) acidified to a pH of 2.5-3.5 *ad libitum*. BBDP and MAD rat colonies at BRM were housed on conventional racks within a VAF barrier facility and either continued to be housed as such once enrolled onto study (BBDP rats) or were transferred to negative-pressure isolators in a separate non-barrier facility (induction of diabetes in MAD rats). Female NOD mice were purchased from The Jackson Laboratory (Stock #001976) at 6 week of age and housed in a dedicated room on ventilated racks within a VAF barrier facility. All animals were observed daily for signs of toxicity such as changes in respiration, changes in motor activity, convulsions, changes in reflexes, cardiovascular signs (redness of skin and/or extremities), piloerection, lethargy and gastrointestinal upset. Any unthrift animal was examined by the study director and/or veterinarian. If the animal appeared to be in unrelieved pain or distress, the animal was euthanized and a necropsy was performed evaluating gross organ size, color and appearance (see assessments below). Atypical findings were sampled and preserved in neutral-buffered formalin for light microscopy studies. Animals were exposed to a 12-hr light/dark light cycle. BRM BBDP and MAD rat colonies were kept at sufficient sizes to ensure adequate numbers of rats to enter onto study in a single enrollment period as described below. NOD mice were purchased from The Jackson Laboratory in pre-study cohorts ranging in size from 100-200 mice; these cohorts were then used to enroll onto studies as described below.

### Ethics Statement

The veterinary care of the animals were in accordance with Test Facility’s standard operating procedures, and regulations outlined in the applicable sections of the Final Rules of the Animal Welfare Act regulations (9 CFR), the *Public Health Service Policy on Humane Care and Use of Laboratory Animals*, the *Guide for the Care and Use of Laboratory Animals*, and the BRM, Inc. Policy on Humane Care [28,29]. The study protocols and any amendments or procedures involving the care or use of animals in studies were reviewed and approved by the Test Facility’s Institutional Animal Care and Use Committee before the initiation of such procedures. The following approved BRM IACUC Docket numbers cover these studies: 06-16, 09-07, 09-23, and 09-30.

### Experimental Procedures

1) For prevention studies using BBDP rats, male rats were selected at 25 or 30 days of age for the DT22669 or CsA study, respectively, and enrolled into groups in a staggered fashion. BBDP rats were administered 0.2 mg/kg CsA or vehicle (intralipid) once daily by IP injection from 30-60 days of age, or 100 mg/kg DT22669 or vehicle (0.9% sodium chloride) once daily by oral gavage for the duration of the study. BBDP rats were monitored for diabetes onset (defined as a positive glycosuria test followed by confirmation with a blood glucose level > 250 mg/dL) three times weekly until 120 days of age. Rats were euthanized within 2 days of diabetes onset or at completion of study.
2) For prevention studies using the inducible MAD rat (formerly the LEW.1WR1 rat), male and female rats 21-25 days of age at initiation of diabetes induction (Day -3). To induce diabetes, each rat was administered 3 µg/g poly (I:C) (Sigma) by IP injection on Days -3, -2, and -1, followed by an IP injection of 1x10^7^ PFU of Kilham rat virus on Day 1. The induction conditions were extensively optimized and reported previously [30,31]. ISO-092 or vehicle was administered via overnight-primed ALZET osmotic pumps implanted on Day -1. As a positive control, a short course of dexamethasone (2 mg/kg, MP Biomedicals) was administered by IP injection on Days 6-10. Pumps were replaced on Day 22. Glycosuria was monitored three times weekly and positive tests were followed up with a blood glucose measurement via handheld glucometer to confirm diabetes onset (≥ 250 mg/dL).
3) For standard late prevention studies with NOD mice, female mice were aged up to 10 weeks of age, when once weekly non-fasted blood glucose measurements were initiated to exclude diabetic mice. Non-diabetic mice with blood glucose levels < 250 mg/dL on Day 1 (12 weeks of age) were enrolled into study and dosed with either 25 or 50 mg/kg ISO-092 or vehicle (50% PBS, 40% PEG, 5% propylene glycol, and 5% polysorbate) by oral gavage once daily for 14 days. NOD mice were monitored for diabetes onset (defined as a non-fasted blood glucose level ≥ 250 mg/dL on two consecutive days) twice weekly until 25 weeks of age. Mice were euthanized within two days of diabetes onset or at completion of study.
4) For GTT late prevention studies with NOD mice, female mice were aged up to 10-12 weeks of age, when once weekly non-fasted blood glucose measurements were initiated to exclude diabetic mice. Non-diabetic mice with blood glucose levels at 14 weeks of age were administered a single IPGTT (2 mg/kg) and mice exhibiting a blood glucose level > 200 mg/dL at either 30 or 60 minutes post challenge and a non-fasted blood glucose level of < 250 mg/dL on Day 1 (1-2 days post IPGTT, Day 1) were assigned to groups and administered the following (in individual studies): 25 mg/kg ISO-092 or vehicle by oral gavage once daily for 14 days; 2 mg/mouse Aralast NP or vehicle (0.9% sodium chloride) by IP injection on Days 1, 4, 7, 10, and 13; or 25 mg/kg Celastrol or vehicle (15% DMSO and 85% cremophor) by oral gavage three times weekly. NOD mice were monitored for diabetes onset (defined as a non-fasted blood glucose level ≥ 250 mg/dL on two consecutive days) twice weekly for up to 90 days. Mice were euthanized within two days of diabetes onset or at completion of study.
5) For reversal studies with NOD mice, female mice were aged up to 10 weeks of age, when once weekly non-fasted blood glucose measurements were initiated to exclude diabetic mice. Beginning at 12 weeks of age until 20 weeks of age, non-fasted blood glucose levels were measured three times weekly and diabetic (defined as a non-fasted blood glucose level ≥ 250 mg/dL on two consecutive days) mice were enrolled into groups in a staggered fashion on the same day as confirmation of diabetes onset (~24 hours post onset, Day 1), and administered the following (in individual studies): 50 µg anti-CD3 F(ab’)2 (clone 145-2C11) or Hamster IgG F(ab’)2 isotype control once daily on Days 1-5 by IP injection ± 1 mg/kg PGC-GLP-1 or PGC control once weekly by SC injection. For these studies the entry criteria was set at blood glucose levels equaling 250-399 mg/dL on two consecutive readings. Mice were euthanized at completion of study or upon reaching a humane end point (non-fasted blood glucose levels ≥ 500 mg/dL for three consecutive measurements performed twice weekly, or clinical signs unresolved after two days of fluid therapy).

## Results

### Model optimization

The program began with an effort to standardize methods and operating procedures. The NOD model is impacted by its environment, and it has been well-known that diabetes incidence rates can vary among animal facilities [32]. We purchased female animals for every experiment directly from The Jackson Laboratory, Bar Harbor. BRM, Inc., did not establish a separate NOD mouse breeding facility. We report that diabetes incidence rates at BRM were comparable to the Jackson Lab’s Bar Harbor facility rates throughout the term of the testing program, with the following exceptions. In 2006, The Jackson Laboratory, Bar Harbor, was undergoing some construction and during that time T1D incidence in their colony was reduced compared to the incidence at the BRM facility. Similarly, in 2010, T1D incidence was reduced at BRM and this also coincided with local construction activities.

Management of glycemic control in subjects enrolled in clinical trials is a very important variable which could impact a trial’s outcome. As a result, in rigorous T1D clinical trials, treatment assignments are double-blinded, and diabetic care is monitored carefully throughout the study. We reasoned that we should also take care to provide the best possible control of glycemia for animals in studies. This proved to be difficult in rodents because formulations of human insulin observed to be long-acting in humans, were not long-acting in rodents [27], and even with multiple daily injections animals experienced wide glycemic excursions and higher overall blood glucose levels. Therefore, we established conditions of control in the NOD using osmotic pumps that provided continuous insulin and kept blood glucose levels low over the course of the treatment. A drawback of this approach was that animals could become hypoglycemic or on the low end of the normal range at higher frequencies than was observed when injections were used.

Since clinical trials for the prevention of type 1 diabetes in humans rely upon determinations of risk, we also performed experiments to measure the progression of pathogenesis metabolically in mice. All NOD mice have an identical genetic predisposition to disease, but only 80-90% succumb, and the disease can develop in different animals at different times. To identify animals with incipient disease, we performed a series of glucose tolerance tests on animals between 12 and 16 weeks age. We determined that impaired glucose tolerance test results (even when random glucose tests were in the normal range) in 14 week old animals is useful to identify those who will progress to T1D within 3-4 weeks after testing (manuscript in revision). This test was used to determine whether agents could prevent the disease at later stages of pathogenesis (pre-diabetic phase) and also to avoid the confounding influences of overtreatment with exogenous insulin therapy.

### Specific Agents Tested

**Figure 1:**
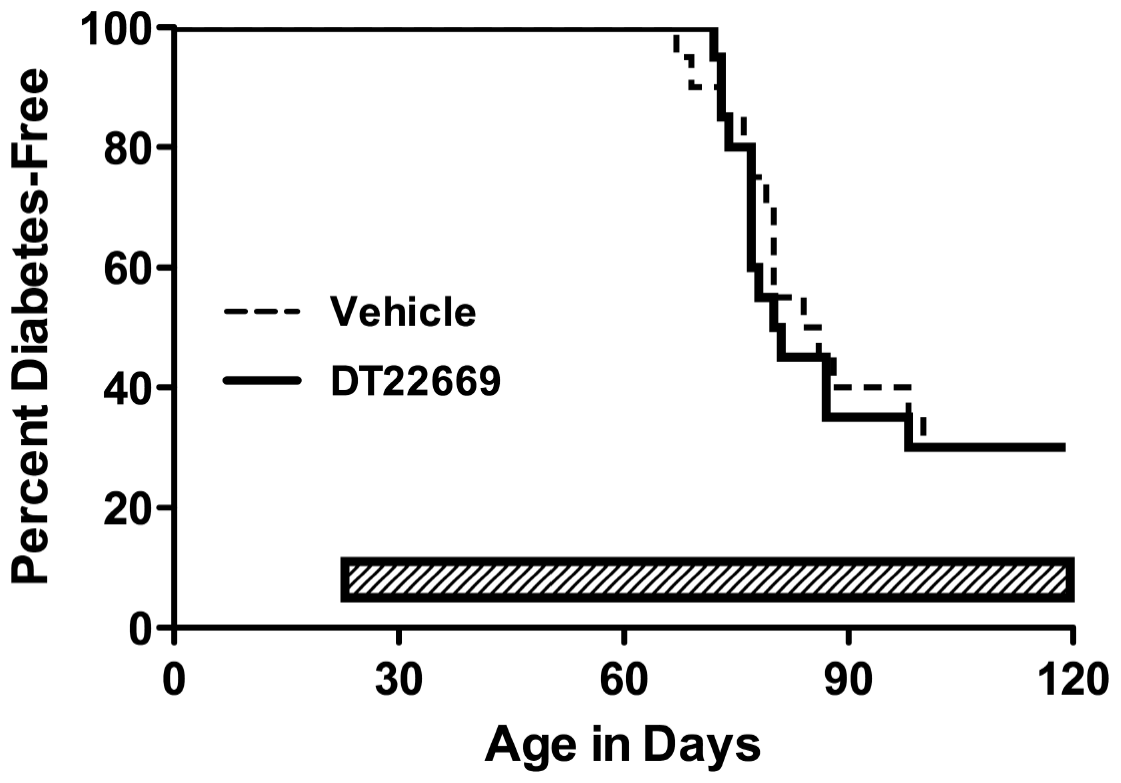
DT22669 does not prevent diabetes in BBDP rats. Male BBDP rats (n=20-24/group) were administered DT22669 or vehicle by oral gavage beginning at 25 days of age until either 120 days of age or diabetes onset.

**Figure 2:**
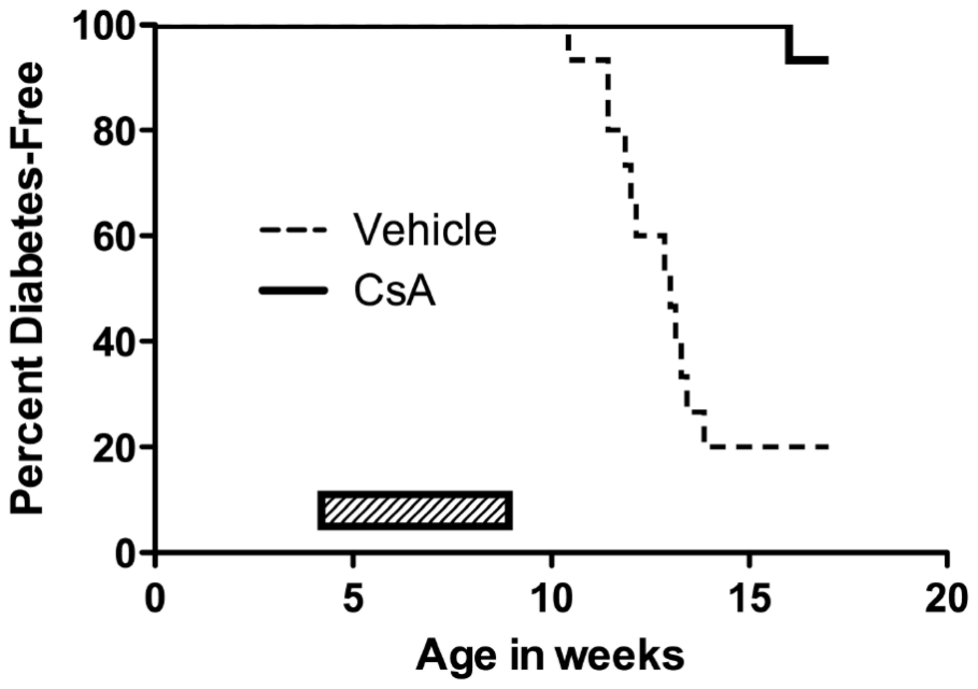
CsA prevents diabetes in BBDP rats. Male BBDP rats (n=20-24/group) were administered CsA or vehicle once daily for 30 days (*p* < 0.0001, Log-rank [Mantel-Cox] test).

**Figure 3:**
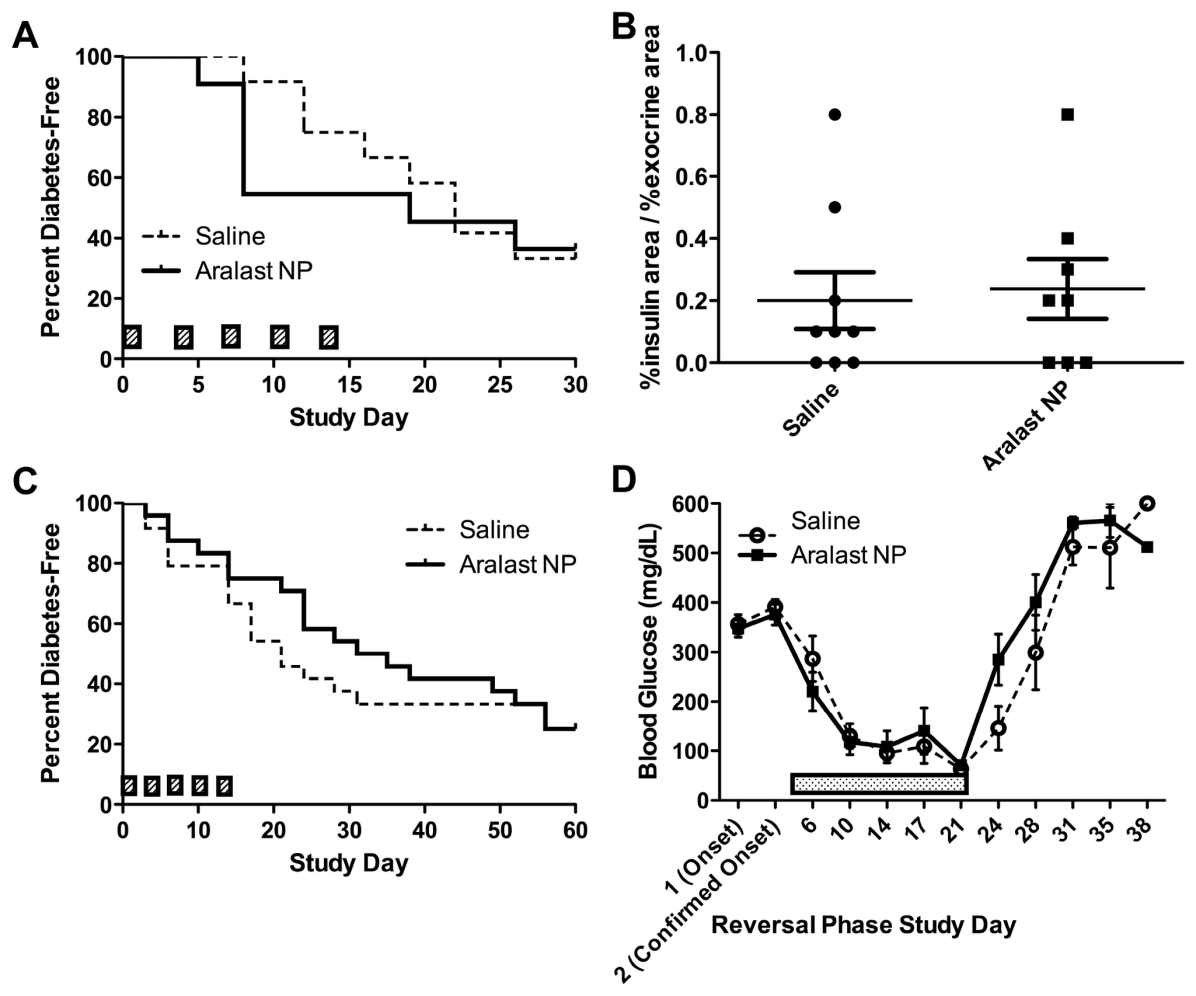
Aralast does not reverse diabetes in NOD mice, nor does it prevent diabetes in dysglycemic NOD mice. (**A**, **C**) Two independent (different cohorts of mice and different lots of reagents) studies were performed with non-diabetic female NOD mice with impaired glucose tolerance at 14 weeks of age (n=11-24/group) administered either 2 mg/mouse Aralast NP or saline on Days 1, 4, 7, 10, and 13. (**B**) Aralast NP did not improve fractional insulin area in A. (**D**) Mice in C that became diabetic continued to receive either Aralast NP saline and were implanted with an ALZET osmotic pumps administering 0.25 U/day insulin (dotted bar). Completion of the original dosing regimen did not lead to remission after cessation of insulin administration at approximately Day 21 post onset.

**Figure 4:**
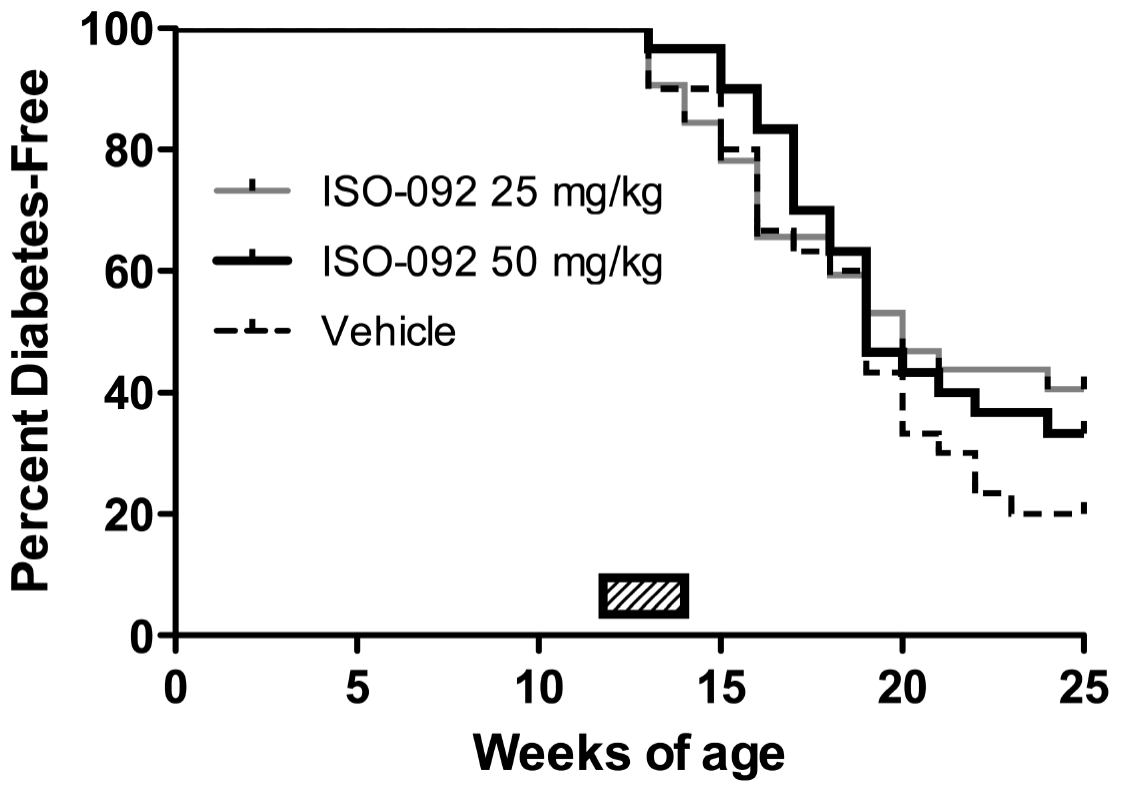
ISO-092 does not prevent diabetes in NOD mice. Non-diabetic female NOD mice (n=33-34/group) at 12 weeks of age were administered ISO-092 or vehicle once daily by oral gavage for up to 14 days or diabetes onset.

**Figure 5:**
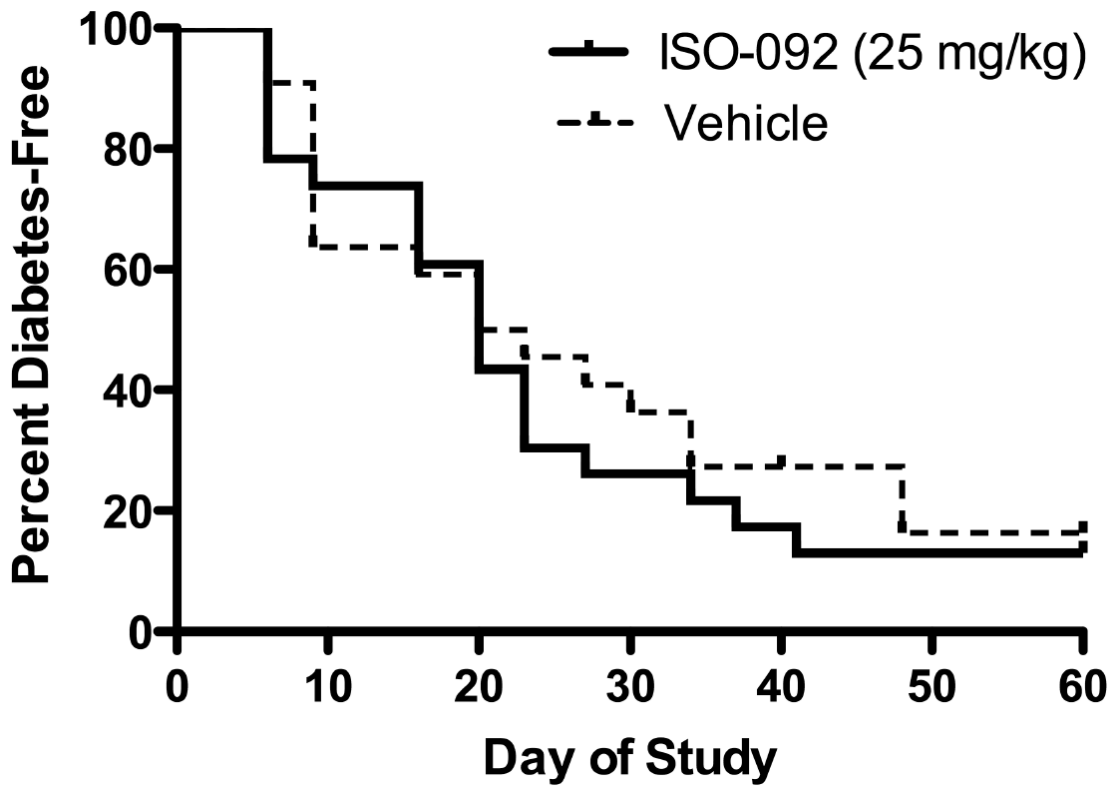
ISO-092 does not prevent diabetes in dysglycemic NOD mice. Non-diabetic female NOD mice with impaired glucose tolerance (n=24/group) at 14 weeks of age were administered ISO-092 or vehicle once daily by oral gavage for up to 14 days or diabetes onset.

**Figure 6:**
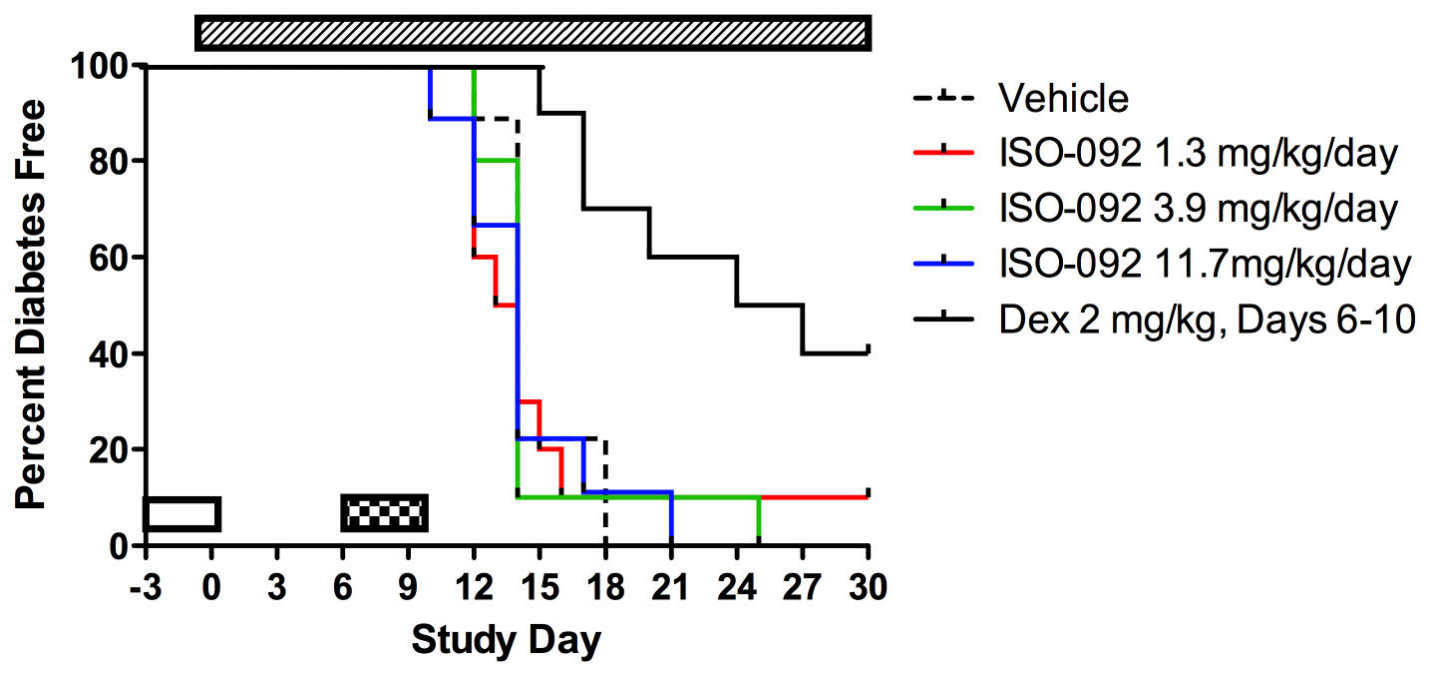
ISO-092 does not block diabetes induction in MAD rats. Male and female MAD (LEW.1WR1) rats (n=10/group) 21-25 days of age on Day -3 were induced for T1D with poly (I:C) on Days -3, -2, and -1 and infected with KRV on Day 1 (clear bar). ISO-092 or vehicle was administered via ALZET osmotic pump beginning of Day -1 (hashed bar). A positive control group was administered dexamethasone once daily by IP injection on Days 6-10 (checkered bar, *p* = 0.0006, vehicle vs. dexamethasone, Log-rank [Mantel-Cox] test).

**Figure 7:**
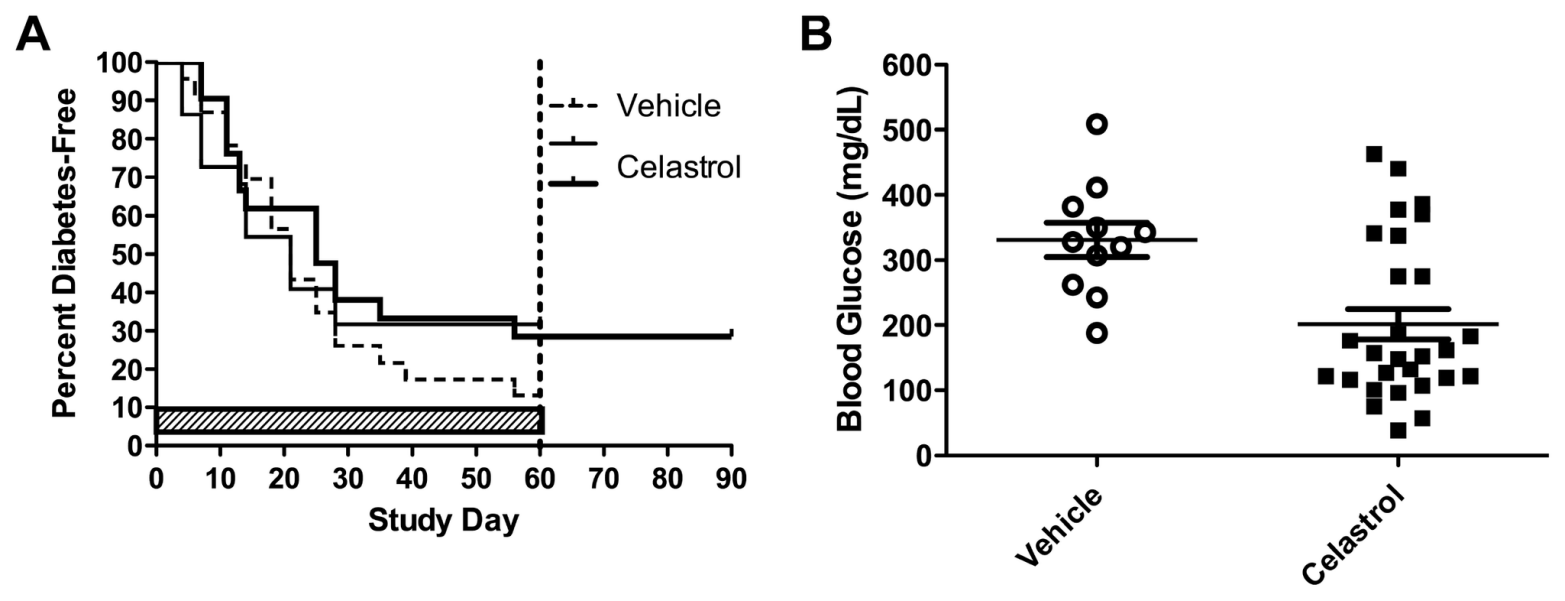
Celastrol does not prevent diabetes in NOD mice, but transiently lowers blood glucose. Non-diabetic Female NOD mice with impaired glucose tolerance at 14 weeks of age (n=24/group) were administered either 25 mg/kg Celastrol or vehicle 3x weekly by oral gavage for up to 60 days. Cessation of dosing (indicated by a verticle dotted line at Day 60) with an additional 30 days of diabetes monitoring in one group did not produce any additional diabetic mice (left panel). Celastrol-treated mice exhibiting blood glucose levels of ≥ 250 mg/dL and dosed later the same day had significantly reduced blood glucose levels the following day during the scheduled measurement to confirm diabetes onset compared to retests from vehicle-treated mice (*p* = 0.0029, unpaired t test, right panel).

1) *DT22669* (*STAT4 inhibitor*) *–*DiaKine Therapeutics’ agent DT22669, one of several lead compounds designed to be an orally available analog of Lisofylline was selected for testing in the BBDP rat model. Unpublished data submitted in the request demonstrated DT22669 was 1.5 times more effective as an inhibitor of IL-12 signaling than LSF, mimicked LSF insulin-stimulatory effect in human islet cells in the presence of a cocktail of cytokines as well as in the presence of IL-12 alone, restored MTT metabolism, suppressed STAT4 phosphorylation in isolated treated NOD splenocytes *in vivo*, and prevented T1D in the NOD mouse. Previous studies had shown that LSF was anti-inflammatory, reducing expression of cytokines including IL-1β, TNF-α, IFN-γ, among other effects [33,34,35]. Previous studies had also shown effects of LSF in preventing and reversing type 1 diabetes in the NOD mouse when used in combination with exendin [36].
BBDP rats were dosed with DT22669 IP at 50mg/kg (dose selected based on preliminary studies), or 100 mg/kg orally, beginning at 25 days of age, and rats were followed for up to 120 days of age. Prior to dose administration, animals were allocated to one of four groups by sequential assignment; the groups were A), IP saline injection (n=20), B) oral saline gavage (n=20), C) IP DT22669 (n=24) and D) oral gavage DT 22669 (n=24). The IP injection arms were discontinued due to the injection-related peritonitis. Peritonitis could have resulted from one or more of the following: injection errors, high pH of injected material, or lack of sterility of the drug solution. No adverse effects were observed in animals treated by oral gavage. However, there was no evidence of efficacy for diabetes prevention by DT 22669 (Figure 1). We did observe clear diabetes prevention in a similar cohort of BBDP rats by cyclosporin A treatment (Figure 2). Thus, we demonstrated that diabetes can be efficiently prevented in the BBDP rat within our facility, using standard diabetes prevention methods [37]. It should be noted that the scope of these studies did not include the evaluation of DT22669 blood levels or target inhibition.

2) *Aralast NP* (*alpha_1_ anti-trypsin*) – alpha_1_ anti-trypsin (or alpha_1_ proteinase inhibitor) is a drug approved for use as a replacement therapy for patients with alpha_1_ anti-trypsin deficiency. The drug has broadly acting anti-inflammatory properties [38]. Several previous studies have shown that this drug can prevent or reverse T1D in the NOD mouse model [39,40,41]. Based on this prior work, the Immune Tolerance Network approved a clinical trial of AAT in type 1 diabetes, but requested an independent test of AAT in the NOD mouse.
A series of small safety studies were performed to ensure that Aralast NP at doses of up to 3 mg/animal was safe in NOD mice, and did not induce anaphylaxis or other adverse events. No adverse events were observed. Then an NOD reversal study was performed using Aralast NP at 3 mg/injection every three days for five total injections with co-administration of either once daily injections of PZI insulin (as needed based on glucose levels) or Humulin R insulin delivered via implanted osmotic pumps. Animals were selected for the study based on two sequential daily random blood glucose tests in the diabetic range, but there was no upper limit of hyperglycemia imposed for this study, and approximately half of the diabetic animals were held for up to 7 days before receiving their first dose of drug or vehicle (for convenience since drug was prepared fresh for daily injections). Control groups were diabetic animals treated with drug vehicle, and matched insulin treatment regimens. The endpoint for the study was diabetes reversal as defined by persistent reading of glucose levels under 250 mg/dL without insulin injections or after pump removal. The study included 14 animals per group and was powered to detect a 30% difference in the incidence of diabetes reversal in drug versus vehicle groups. Regardless of the insulin treatment regimen used, Aralast NP did not reverse recent onset T1D (data from the osmotic pump study are shown in Figure 3). Since previous work suggested that Aralast might be ineffective if used after animals had experienced very high blood sugars (above 350 mg/dL, Koulmanda, personal communication.), we also tested whether Aralast NP was able to prevent type 1 diabetes. For this study, we enrolled animals with values in the “impaired” but non-diabetic range on a glucose tolerance test performed at 14 weeks of age. These animals were also tested daily and none were in the diabetic range at the time of treatment. Aralast NP was unable to prevent progression to frank diabetes, in animals with abnormal glucose tolerance in two separate studies (Figure 3).

3) *ISO-092* (*MIF inhibitor*) – Testing of a potent MIF inhibitor was proposed by Tom Coleman and Yousef Al-Abed at the Feinstein Institute for Medical Research in Manhasset, New York. The small molecule MIF inhibitor ISO-092 is a fluorinated analog of ISO-1 with oral bioavailability [42]. MIF has been shown to enhance beta cell destruction in mice [43] and *to increase diabetes incidence in NOD mice* [44].
Non-diabetic NOD mice at 12 weeks of age were treated daily for 14 days or until diabetes onset with 25mg/kg, or drug vehicle by oral gavage (standard late prevention model). Pilot studies had indicated that the drug was well-tolerated, and no adverse events were observed in this study. Animals (n=33-35 per group, having an 80% power to detect a 50% reduction in diabetes incidence) were observed for diabetes onset through 25 weeks of age. The agent provided no protection from progression to diabetes (Figure 4).
NOD mice at 14 weeks of age and with abnormal glucose tolerance, were treated daily for 14 days with ISO 92 25mg/kg, or drug vehicle, by oral gavage. Pilot studies had indicated that the drug was well-tolerated, and no adverse events were observed in this study. Animals (n=23 per group, having an 80% power to detect a 50% reduction in diabetes incidence) were observed for diabetes onset through 25 weeks of age. The agent provided no protection from progression to diabetes (Figure 5). Subsequent PK studies performed by BRM showed that ISO-092 had a half-life *in vivo* of approximately 1 hour whether administered via IP or PO injection, suggesting that one cause of lack of efficacy could have been inadequate dosing.
A follow-up study using osmotic pumps to deliver a continuous dose of ISO-092 at 1.3, 3.9, or 11.7 mg/kg/day was performed. For this study, the KRV and poly (I:C) inducible diabetes model in MAD rats was used. In agreement with earlier work, dexamethasone treatment could prevent diabetes in this model [31]; however, continuously delivered IS0-092 at multiple concentrations did not (Figure 6).

4) *Cis-Tetracosenoyl Sulfatide* – Vipin Kumar of Torrey Pines Institute for Molecular Studies requested that we test a synthetic sulfatide (cis-tetracosenoyl) known to activate CD1d-restricted type II NKT cells and impact T1D by regulating type I NKT cells. Prior studies had shown that treatment of animals with brain-derived sulfatide (ceramide galactoside-3-sulfate) as well as synthetic long chain sulfatides could protect against experimental autoimmune encephalomyelitis and a model of induced autoimmune hepatitis, as well as reducing incidence of T1D in the NOD mouse [45]. Since there was significant preliminary data on acute effects of drug treatment in the NOD mouse, we decided to test our dosing regimen. Two pilot studies were performed in which the synthetic sulfatide or the brain derived mixture was injected into mice and IL-12 and IFNμ production were measured. C57Bl/6 mice were used since that was the strain which had been shown to previously respond to the drug [46]. However, in neither study were IFN-γ and IL-12 produced in response to the injection of sulfatide products, in contrast to what was previously published. As a result, no further experiments using these drugs were performed, but further studies in the requestor’s lab confirmed that the sulfatide preparations used showed reduced biological potency in other studies (V. Kumar, personal communication).
5) *Celastrol* – Celastrol is a natural triterpene compound isolated from *Tripterygium* wilfordii, with *in vitro* and *in vivo* anti-inflammatory properties, treating rodent models of arthritis [47] as well as anti-cancer properties, and has been shown to impact hematopoiesis in the mouse [48]. Preliminary data from the requestor suggested that celastrol might reduce the incidence of T1D in the NOD mouse. We tested celastrol for prevention in mice with impaired glucose tolerance. The study was powered to detect a 50% reduction in T1D incidence in the active as compared to the control group (n=24 animals in each group). The drug was dosed at 25mg/kg 3 times per week through 60 days, by oral gavage. Animals were followed for 61 days, except for an additional group which was followed for 91 days. Although there was a slight reduction in diabetes incidence in the treated group up to 61 days (16% reduction), this difference was not statistically significant (Figure 7). Of interest, blood glucose levels in Celastrol-treated mice were found to be slightly reduced on the day after dosing, but not at two days post dosing; suggesting that celastrol lowers blood glucose levels acutely (Figure 7). In addition, an increased incidence of hypoglyemic events were observed in the pre-diabetic Celastrol groups as compared to the controls, with 8 total events noted in the Celastrol groups and none in the control group. Although we cannot determine whether the differences are statistically significant, these results are consistent with an acute blood glucose lowering effect of Celastrol.
6) *Tregitopes* – The biotech company EpiVax has identified a set of natural, human regulatory T cell epitopes (“Tregitopes”) present in Fc and Fab domains of IgG that may be responsible for tolerance to idiotypic epitopes. When incubated with peripheral blood mononuclear cells (PBMCs) in vitro, CD4+ T cells that are specific for Tregitopes increase CD25/Foxp3 expression, proliferate, and increase expression of regulatory cytokines and chemokines [49]. The mechanisms of action and applications for Tregitopes have been evaluated by more than five collaborating laboratories over a range of models that include autoimmunity, allergic airway disease (OVA), and in a standard model immunogenicity (D011.10, unpublished data). Additional in vivo studies have been performed in transplantation (mixed lymphocyte reaction, cardiac transplant), and gene therapy [50]. In these prior studies, Tregitopes co-administered with proteins were observed to suppress antigen-specific T cell and antibody responses, and induce Treg expansion and function.

**Figure 8:**
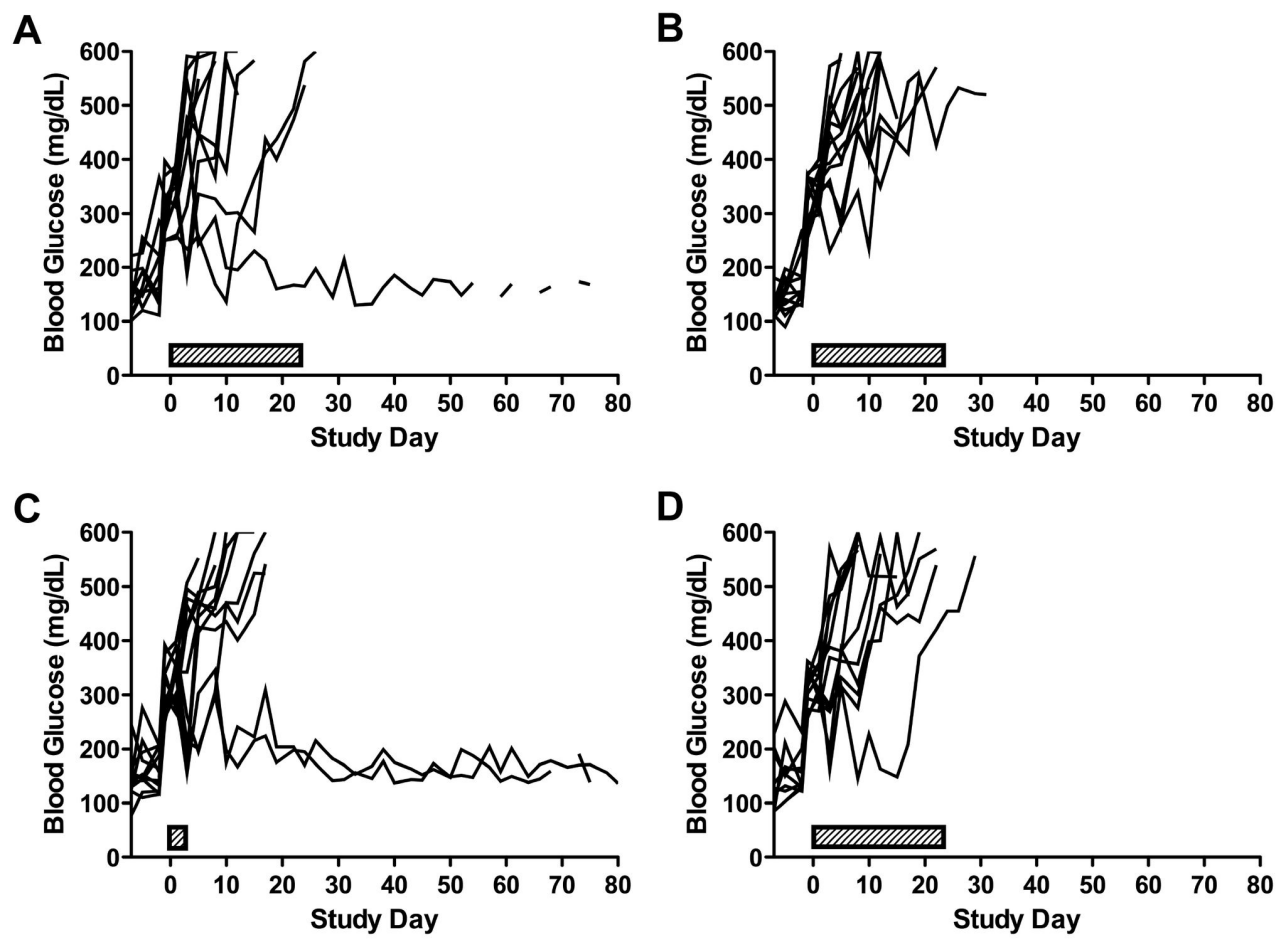
Rare remissions observed in diabetic NOD mice treated with Tregitopes. Female NOD mice were screened for diabetes onset (defined as non-fasted blood glucose levels ≥ 250 mg/dL on two consecutive days) between 12–20 weeks of age 3x weekly, enrolled into study groups in a staggered fashion, and initiated dosing on the day of confirmed onset (Day 1). (**A**) Tregitopes (100 µg/mouse) formulated in liposomes and dosed once weekly for four weeks beginning on Day 1 by IP injection resulted in 1 out of 12 mice exhibiting stable remission (defined as non-fasted blood glucose levels < 200 mg/dL). (**B**) Irrelevant peptide (100 µg/mouse) formulated in liposomes and dosed as in A did not result in remission for any of the 12 mice in that group. (**C**) Tregitopes (100 µg/mouse) formulated in IFA and dosed once on Day 1 resulted in 2 out of 12 mice exhibiting stable remission. (**D**) Tregitopes (100 µµg/mouse) plus preproinsulin peptides (50 µg/mouse) formulated with liposomes and dosed as in A did not result in any of the 12 mice in that group exhibiting stable remission.

The company provided preliminary data (accepted for publication in Experimental Diabetes Research) that showed that Tregitopes could be effective for reversing T1D in NOD mice. On the basis of the preliminary data, we tested Tregitopes supplied by EpiVax in a diabetes reversal study, which was powered (80%, 0.05, two-tailed) to be able to detect a 50% increase in remission between the active treatment and the control groups. A spontaneous remission rate was assumed at 1 in 100 animals and was validated in the course of these studies. There were 8 testing groups, with n=12 new onset diabetic mice (i.e., exhibiting non-fasted random blood glucose levels of 250-399 mg/dL on two consecutive days) per group. Enrolled mice received the initial dose administration ~24 hours after diabetes onset (the first of the two blood glucose levels 250-399 mg/dL). Groups were, 1) Liposomes, 2) murine preproinsulin peptides (mPPI) plus liposomes, 3) mPPI plus murine Tregitopes (mTregitopes) plus liposomes, 4) mPPI, plus irrelevant peptides plus liposomes, 5) mTregitopes plus liposomes, 6) irrelevant peptides plus liposomes, 7) vehicle, 8) mTregitopes plus IFA. Three total mice remitted, 1 of 12 in the mTregitope + liposome group (5) and 2 of 12 in the mTregitope + IFA group (Figure 8). Remission was defined as restoration and maintenance of normoglycemia (defined as non-fasted blood glucose levels < 200 mg/dL) for ≥ 80 Days. There were no statistically significant differences between the groups, but there were some notable trends. The appearance of remitted animals in the groups treated with Tregitopes was promising. Interestingly, within the 5 control groups combined there were 9 mice (15%) that rapidly progressed and required euthanasia in the first week of treatment as compared to 3 such mice (8%) in the combined treatment groups.

**Figure 9:**
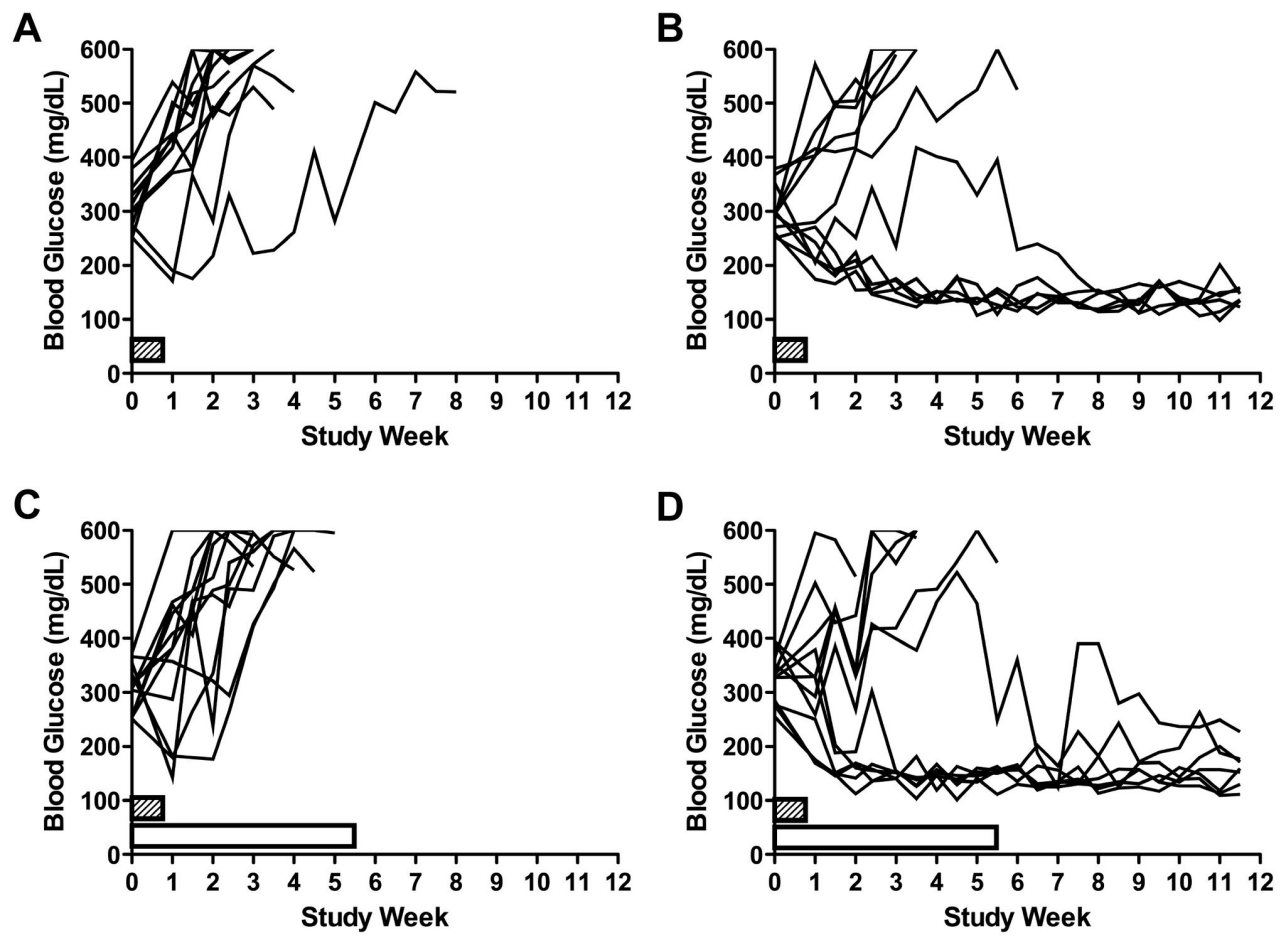
Anti-CD3 antibody treatment induced remission in diabetic NOD mice, but remission was not enhanced by PGC-GLP-1. Female NOD mice were screened for diabetes onset (defined as non-fasted blood glucose levels ≥ 250 mg/dL on two consecutive days) between 12–20 weeks of age 3x weekly, enrolled into study groups in a staggered fashion and initiated dosing on the day of confirmed onset (Day 1). (**A**) Hamster IgG F(ab’)2 control antibody-treated mice did not enter remission when dosed at 50 µµg/mouse on Days 1-5 by IP injection, whereas administration of anti-mouse CD3 (145-2C11) administered alone and dosed as in A resulted in 6 out of 12 mice entering stable remission (defined as non-fasted blood glucose levels < 200 mg/dL) (**B**). None of the 12 PGC-GLP-1-treated mice entered remission when dosed at 1 mg/kg once weekly by SC injection in the presence of control antibody (**C**). PGC-GLP-1, when administered as in C did not have an effect on the frequency of remission in the presence of anti-CD3 (**D**) compared to B.

7) *PGC-GLP-1* – Glucagon-like peptide 1 (GLP-1) is an intestinal hormone that increases insulin secretion in response to a meal, by a number of different mechanisms [51,52]. GLP-1 has been approved for use in type 2 diabetes, based on its efficacy in helping reduce blood glucose. Published studies have also suggested that GLP-1 can promote beta cell replication in rodents [53]. For these reasons, GLP-1 is an interesting molecule for the treatment of type 1 diabetes, but the native peptide has a very short half-life. The company PharmaIN has non-covalently coupled GLP-1 to a nanocarrier (PGC) and proposed that this longer-lived compound be tested in the NOD T1D model. The PGC nanocarrier molecules significantly slows the digestion of GLP-1 by dipeptidylpeptidase-IV (DPP-IV) and decreases GLP-1 glomerular filtration, thus prolonging GLP-1’s half-life in the circulation [54]. Preliminary data from the company suggested that PGC-GLP-1 given at 3mg/kg/week for 8 weeks starting at four weeks of age could reduce the incidence of T1D in the NOD so the hypothesis that PGC-GLP-1 could possibly promote diabetes reversal in newly onset diabetic NOD mice when used in combination with anti-CD3 was tested. We performed a 6 group, n=12 mice/group reversal study designed to detect a 35% increase in diabetes remission by PGC-GLP-1 in the treated groups as compared to the vehicle control (80% power, 0.05, two-tailed test). Animals with confirmed diabetes (blood glucose values between 250 and 399 mg/dL) were enrolled into the study. Groups were, PGC (nanocarrier alone) or PGC-GLP-1 (a long-acting GLP-1 peptide non-covalently coupled to a nanocarrier, 1mg GLP-1/kg/week) co-administered with an immunomodulator (anti-CD3, clone 145-2c11, F[ab’]2) or control F(ab’)2 antibody. The number of mice exhibiting normoglycemia/total number of mice enrolled was as follows: Group 1 (Control IgG) = 0/10; Group 2 (anti-CD3) = 6/12; Group 3 (PGC + control IgG) = 1/10; Group 4 (PGC + anti-CD3) = 8/12; Group 5 (PGC-GLP-1 + control IgG) = 0/12; and Group 6 (PGC-GLP-1 + anti-CD3) = 6/12 (Figure 9). PGC-GLP-1 did not demonstrate efficacy at 1mg GLP-1/kg/week either alone or in combination with anti-CD3 in this model. The remission frequency observed herein (50%) for anti-CD3 was very similar to that previously reported (56% [55]). Thus, we demonstrated that diabetes can be efficiently reversed in the NOD mouse within our facility, using a method which is well established in many laboratories [7,56,57,58,59].

## Discussion

We report here the efforts of the NIDDK to coordinate voluntary standardized testing of different treatments for T1D that are under development by academic investigators and small companies, using rodent models of disease and carried out by an independent contract research laboratory. We used internally consistent methods, and attempted a thorough reporting of study results and procedures, following some of the principles outlined by Landis and colleagues for preclinical research [26]. Our goals were to provide an opportunity to test novel compounds for possible therapeutic application in T1D. We used an open solicitation for ideas and compounds, and offered access to requestors whose agents were selected for study, to have input on the experimental design of studies, with the caveat that the studies had to be performed in the BRM contract laboratory using standard operating procedures whenever possible. Our goal was not to try to exactly reproduce any studies previously performed, but rather to extend the testing in an independent laboratory, using methods that were modeled as closely as possible on the human clinical trial experience. For example, we used glucose tolerance testing to screen NOD mice for “late prevention” studies, modeled on Type 1 Diabetes TrialNet’s approach to risk screening [60,61]. Overall, the performance of the contract laboratory was sufficient to accomplish most of the testing goals. The laboratory also had the capability to perform mechanistic and pharmacokinetic studies for those agents that showed preliminary efficacy. Mechanistic studies were rarely done in advance of a demonstration of preclinical efficacy.

Although it would have been preferable, drug dosing and pharmacokinetics could not be evaluated for every agent tested in the program. In most cases, since we selected drugs that had shown some prior efficacy, we used doses that had been shown previously to be effective. In one case, dosing and effects of drug were tested in a pilot prior to efficacy testing, because of a change in drug manufacturing (Kumar, personal communication). As reported here, the drug did not induce the expected acute effects, and further studies confirmed that the drug was not as active as expected (Kumar, personal communication). In other cases, drugs supplied by investigators were lead compounds and pharmacokinetic assays were unavailable, so it would not have been possible to determine whether drug failure was due to dosing or bio-availability. Tregitope, for example, is a drug in development for which dose, delivery, and route of administration have yet to be optimized. Any one of these drug attributes, or combinations thereof, could impact the ability to demonstrate efficacy. However, agents such as the Tregitopes were chosen for testing because there was preliminary data that demonstrated at least some prior efficacy in T1D in at least one rodent model, whether or not PK and dosing were optimized. When an agent had no prior testing in a T1D model, but for which PK was available, we did perform a dosing PK experiment (for example, ISO-92).

This program also illustrated some of the challenges in preclinical testing. It turned out to be a challenge to get agents into the program for testing. Some companies were hesitant to supply agents, given the expectation that all results, including negative results, would be made public. Some academic researchers were interested in the program initially, but declined to proceed with testing when they learned that they would not receive funds to perform the testing in their own laboratory under their own direction. We found that negotiation of a materials transfer agreement could be a barrier to participation for some companies, but progress was made on developing a document that NIDDK and most collaborators could accept (http://www.t1diabetes.nih.gov/T1D-PTP/NIDDKT1D-PTPIncomingMTA.pdf).

The methods presented here are an improvement in the state of the art, but our results also point to further opportunities for improvement. For example, adding standard pharmacokinetic and mechanism of action biomarker testing would improve the interpretation of negative efficacy results. Improved preclinical testing does not always equate to standardization and optimization of methods, although those things are important. Preclinical testing results will perhaps be most robust when similar results are reported from different laboratories, using different models, and different but rigorous methods, all thoroughly reported. Since rodent models of type 1 diabetes mimic many of the pathogenic processes involved in disease progression in humans, they remain an essential step for the proof of concept for all the novel therapies. Better preclinical testing will deliver benefits to researchers studying disease process, drug developers testing candidate treatments, and regulators/funders seeking to establish potential efficacy in rodent models, all with the goal to improve the efficiency, safety, and outcomes of clinical trials in type 1 diabetes.
